# Information Geometry, Complexity Measures and Data Analysis

**DOI:** 10.3390/e24121797

**Published:** 2022-12-09

**Authors:** José M. Amigó, Piergiulio Tempesta

**Affiliations:** 1 Centro de Investigación Operativa, Universidad Miguel Hernández, 03202 Elche, Spain; 2 Departamento de Física Teórica, Facultad de Ciencias Físicas, Universidad Complutense, 28040 Madrid, Spain; 3 Instituto de Ciencias Matemáticas, C/ Nicolás Cabrera, No 13–15, 28049 Madrid, Spain

In the last several years, a new approach to information theory, called information geometry, has emerged [1,2]. Its main objective is the investigation of the geometrical structures that can be introduced in the manifold and are associated with the set of probability distributions of a statistical model. In this approach, one defines a Riemannian metric in a manifold of probability distributions, together with dually coupled affine connections. Information geometry provides a new methodology applicable to various areas of information sciences, such as statistical inference, quantum information theory, machine learning, convex optimization and time-series analysis. It is also a key tool for other areas, such as neurocomputing (where a set of neural networks form a neuro-manifold, a nonlinear system equipped with the Fisher metric).

Complexity measure is a broad concept that embraces any way of characterizing a process or a generic output according to its “complexity” in a predefined sense. Familiar examples include (i) Lempel–Ziv complexity and Shannon entropy in the case of sequences with discrete alphabets, (ii) differential entropy and permutation entropy in the case of analog signals and (iii) Kolmogorov entropy and topological entropy in the case of measure-preserving or continuous dynamics. Further examples include other information–theoretical tools (Renyi and Tsallis entropies, as well as a long list of entropy-like measures), statistical tools (statistical complexity) and dynamical tools (recurrence plots, the correlation integral) together with a variety of complexity–causality planes, ordinal networks and ad hoc tools based on symbolic dynamics. Such a multiplicity of approaches attests to the great interest in this topic.

As for data analysis, this is a multidisciplinary topic that is going through a period of intense research, where old and new ideas are driving interesting developments. Thus, most of the complexity measures mentioned above are becoming increasingly popular in nonlinear time-series analysis. At the same time, machine learning tools, such as recurrent nets and reservoir computing, are pushing the limits of time-series prediction further. New synergies are expected to arise from the application of deep learning to dynamical and complex systems.

This Special Issue, “Information Geometry, Complexity Measures and Data Analysis”, aims to showcase recent progress in the intersections between complex systems, information theory and data analysis, which include complexity measures in the form of generalized entropies and dynamical entropies [3]. Information geometry also belongs to this intersection. Nonlinear time-series analysis is a paramount example of data analysis applied to complex systems. These few examples show the ubiquity of the topics encompassed in this Special Issue in the current hard and soft sciences.

Such diversity, both in theoretical issues and in applications, is also reflected in the contributions to this Special Issue, for which a summary is provided below.

In the review [4], Hernández and Amigó describe the key aspects of an important class of learning models, attention mechanisms, which recently emerged as a new paradigm in machine learning. Although traditional deep learning models do not perform sequential reasoning, attention mechanisms allow researchers to focus on a specific set of data, which helps to promote the execution of complex reasoning tasks. In their article, the authors show in detail how attention mechanisms, when combined with other classical tools of machine learning, offer a description of certain fundamental features of complex systems, such as sequential reasoning, long-term dependencies and part integration.

Fisher’s information measure, which plays a crucial role in information geometry, is the key tool used by Kowalski and Plastino in their study [5] on quantum decoherence in a system that accounts for the interaction between matter and a given field. A detailed description of the quantum–classical changeover is obtained in terms of Fisher’s measure; indeed, the quantum–classical transition is interpreted as a process of information gain. This, in turn, provides us with clear insight into the role played by the uncertainty principle in these processes. As a case study, the authors focused on the dynamics generated by a semi-classical Hamiltonian that represents the zero-th mode contribution of a strong external field to the production of charged meson pairs.

Cholewa and Oprocha, in their article, focus on α-limit sets in Lorenz maps [6]. Lorenz maps are interval maps which are realized as Poincaré sections in the Lorenz attractor. These maps have been largely investigated by means of very different techniques, and many results concerning the underlying dynamics are currently available. However, the authors are able to prove the quite unexpected result that α-limit sets in Lorenz maps need not be completely invariant. Analogous results are proved for unimodal maps. The analysis proposed is also used to compute the topological entropy of interval maps in several concrete cases.

Yan et al. [7] deal with an interesting engineering problem: how to identify wind turbine gearbox faults in an effective way. To this aim, the authors designed a new fault diagnosis method based on generalized composite multiscale Lempel–Ziv complexity. The approach proposed is validated by suitable experimental and engineering data analysis, showing its effectiveness and accuracy in the detection of different gear health conditions. Additionally, the authors compare their methodology with other standard ones, such as traditional multiscale Lempel–Ziv complexity, permutation entropy, etc., proving the higher accuracy of their approach.

Liang [8] focuses on describing the interaction between two subsystems of a complex system which per se represent complex systems as well. The relation among subsystems of a larger, parental complex system is investigated by means of a rigorous formalism based on the representation of causal relations in terms of information flow between the considered subsystems. The author obtains analytic formulas, and, under a Gaussian assumption, maximum likelihood estimators, which have been applied to the case of an autoregressive process as a validation procedure. This analysis is potentially relevant in the study of neural processes and in the construction of climate change models.

In [9], Borges, Kodama and Tsallis, starting from the algebraic framework of nonextensive statistical mechanics, propose a q-deformation of some classical number-theoretical notions. More precisely, an interesting construction of q-natural and q-prime numbers is introduced, with which the authors are able to obtain several novel q-zeta functions. They generalize, in different ways, the celebrated Riemann zeta function. Additionally, the corresponding algebraic properties are studied in detail.

Biró et al. [10] introduce the interesting notion of f-gintropy in the realm of econophysics studies. The authors propose using the density of the Gini index on a Lorenz curve, the so-called gintropy, in a more general setting, when the standard income value is replaced by a monotonic function. In this way, it is possible to construct a divergence measure which can efficiently detect wealth inequalities in the income distributions of populations of different countries.

We thank all the authors that have contributed to this Special Issue for their excellent work and timely submission.
